# OpenProt: a more comprehensive guide to explore eukaryotic coding potential and proteomes

**DOI:** 10.1093/nar/gky936

**Published:** 2018-10-09

**Authors:** Marie A Brunet, Mylène Brunelle, Jean-François Lucier, Vivian Delcourt, Maxime Levesque, Frédéric Grenier, Sondos Samandi, Sébastien Leblanc, Jean-David Aguilar, Pascal Dufour, Jean-Francois Jacques, Isabelle Fournier, Aida Ouangraoua, Michelle S Scott, François-Michel Boisvert, Xavier Roucou

**Affiliations:** 1 Department of Biochemistry, Université de Sherbrooke, Sherbrooke, Québec, Canada; 2 PROTEO, Quebec Network for Research on Protein Function, Structure, and Engineering, Université de Lille, F-59000 Lille, France; 3 Center for Computational Science, Université de Sherbrooke, Sherbrooke, Québec, Canada; 4 Biology Department, Université de Sherbrooke, Sherbrooke, Québec, Canada; 5 INSERM U1192, Laboratoire Protéomique, Réponse Inflammatoire & Spectrométrie de Masse (PRISM), Université de Lille, F-59000 Lille, France; 6 Informatics Department, Université de Sherbrooke, Sherbrooke, Québec, Canada; 7 Anatomy and Cell Biology Department, Université de Sherbrooke, Sherbrooke, Québec, Canada

## Abstract

Advances in proteomics and sequencing have highlighted many non-annotated open reading frames (ORFs) in eukaryotic genomes. Genome annotations, cornerstones of today's research, mostly rely on protein prior knowledge and on *ab initio* prediction algorithms. Such algorithms notably enforce an arbitrary criterion of one coding sequence (CDS) per transcript, leading to a substantial underestimation of the coding potential of eukaryotes. Here, we present OpenProt, the first database fully endorsing a polycistronic model of eukaryotic genomes to date. OpenProt contains all possible ORFs longer than 30 codons across 10 species, and cumulates supporting evidence such as protein conservation, translation and expression. OpenProt annotates all known proteins (RefProts), novel predicted isoforms (Isoforms) and novel predicted proteins from alternative ORFs (AltProts). It incorporates cutting-edge algorithms to evaluate protein orthology and re-interrogate publicly available ribosome profiling and mass spectrometry datasets, supporting the annotation of thousands of predicted ORFs. The constantly growing database currently cumulates evidence from 87 ribosome profiling and 114 mass spectrometry studies from several species, tissues and cell lines. All data is freely available and downloadable from a web platform (www.openprot.org) supporting a genome browser and advanced queries for each species. Thus, OpenProt enables a more comprehensive landscape of eukaryotic genomes’ coding potential.

## INTRODUCTION

An ever-increasing number of studies relate the discovery of functional yet non-annotated open reading frames (ORFs) across eukaryotic genomes (1–8). These are usually small ORFs encoded in currently annotated non-coding RNAs (ncRNAs) (9–11). However, a substantial number are present in mRNAs, either overlapping the CDS or within the 5′ or 3′ ‘untranslated’ regions (UTRs) (6,12–17). They have been found involved in numerous cellular functions, from calcium or insulin regulation to mitochondrial biogenesis (6,7,10,11,16). These examples highlight both the underestimation of coding potential in eukaryotic genomes relayed by current annotations, and the polycistronic nature of eukaryotic genes (6). Since genome annotations lay the foundation for proteomics and sequencing explorations, such underestimation has consequences on most of today's research.

Recent efforts for a more comprehensive view of eukaryotic genomes’ coding potential have focused on annotation of small ORFs, defined as any ORF between 10 and 100 codons, alongside associated evidence from conservation, ribosome profiling and/or mass spectrometry (18–20). Yet, these databases suffer limitations, notably a maximum length threshold that forbids detection of ORFs longer than 100 codons, and they do not account for the polycistronic nature of eukaryotic genomes. In parallel, proteogenomics strategies are emerging to offer an unbiased approach to the study of eukaryotic proteomes, yet they remain the expertise of a few and still depend on sample preparation adapted to the identification of small proteins (21–24). Despite these significant studies, we still lack a systematic approach to fathom the deepest parts of eukaryotic proteomes.

Here, we present OpenProt (www.openprot.org), the first database upholding a polycistronic model of eukaryotic genes to date. OpenProt distinguishes three ORF categories: already annotated ones (RefProts), novel RefORF isoforms (Isoforms, II_ accessions) and novel alternative ORFs (AltProts, IP_ accessions). We define as AltProt the product of any unannotated ORF, anywhere on transcripts (ncRNAs and mRNAs), that do not display protein sequence similarity with a RefProt from the same gene (otherwise categorized as novel isoform: product from an unannotated ORF with a significant sequence similarity to a RefProt from the same gene). OpenProt currently offers deep annotation for 10 species, cumulating supporting evidence of protein orthology, translation and expression. Moreover, through custom downloads and a user-friendly web platform, OpenProt enables wide applications, making this ‘hidden’ proteome easily accessible to the wider scientific community. OpenProt thus aims to foster discoveries of functional yet currently non annotated proteins.

## MATERIALS AND METHODS

### Open reading frames (ORFs) prediction

**Figure 1.**
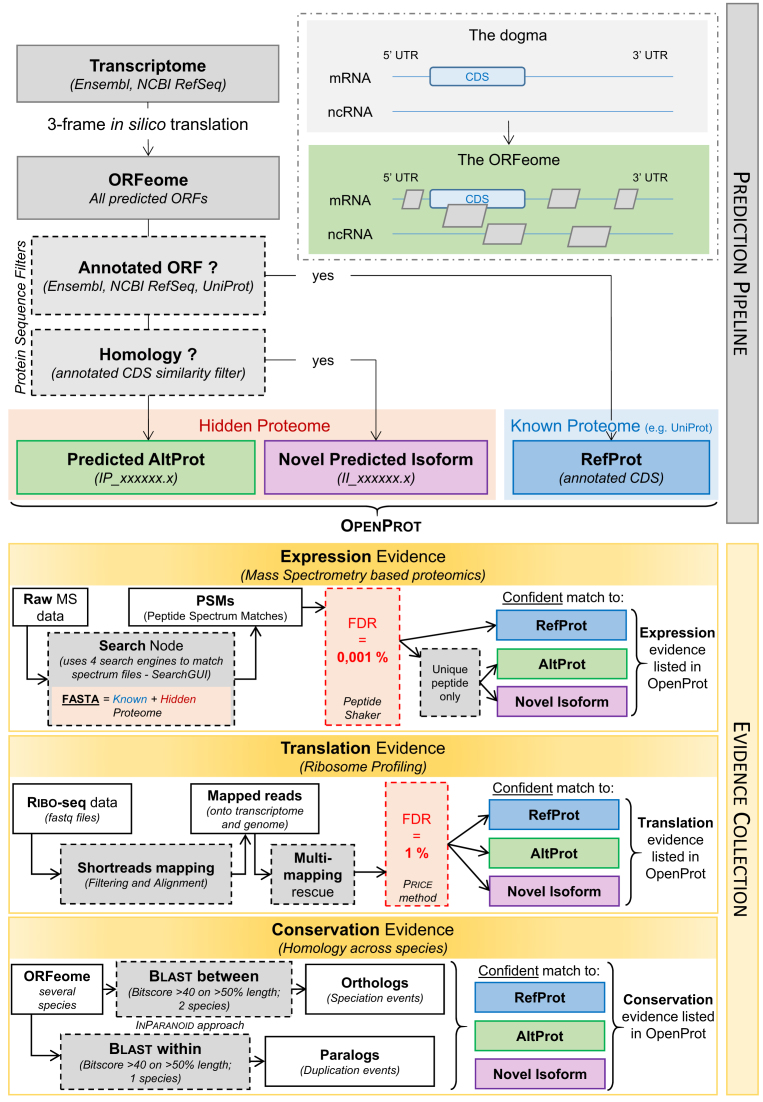
OpenProt pipeline graphical representation. OpenProt pipeline contains two main features: prediction and evidence collection. OpenProt enforces a polycistronic model of eukaryotic genes contrary to the actual dogma of one CDS per transcript. The protein sequence similarity filter (Homology) holds two arguments as described in the material and methods. The hidden proteome consists of currently non-annotated ORFs highlighted by OpenProt. These ORFs are either novel isoforms of known CDS (II_accessions) or novel alternative proteins (IP_ accessions). All evidence collection parameters are described in the material and methods section.

The first step of OpenProt pipeline is the ORF prediction (Figure 1). First, we retrieve an exhaustive transcriptome by combining two well-used annotations (NCBI RefSeq (25) and Ensembl (26)). Annotations overlap is not whole because of variations in algorithms and information sources. In a context of exploration and discovery, a more complex annotation is preferable (27). Hence, we retrieve NCBI RefSeq and Ensembl annotations and compile them into a more exhaustive one. For example in human, NCBI RefSeq (GRCh38.p7) contains 109 077 mRNAs and 29 484 ncRNAs, while Ensembl (GRCh38.83) contains 93 855 mRNAs and 105 150 ncRNAs; only 7578 RNAs are common to both annotations. The source annotation is associated with each ORF prediction so that users can look at predictions from either annotation alone if preferred. Genome assemblies and annotation releases currently supported by OpenProt are listed for each species in Table 1. We then perform a 3-frames *in silico* translation using EMBOSS Transeq (28) to predict all possible ORFs with an ATG start codon and a minimum length of 30 codons. This constitutes the OpenProt ORFeome.

**Table 1.** OpenProt (1.3) prediction pipeline output

		Annotations		ORFeome *(both annotations)*			
Species	Genome assembly	NCBI RefSeq	Ensembl	Total #	Ref #	II_ #	IP_ #
*Homo sapiens*	GRCh38.p5	GRCh38.p7	GRCh38.83	646 403	129 888	55 053	461 462
*Pan troglodytes*	CHIMP2.1.4	CHIMP2.1.4	CHIMP2.1.4.87	227 950	37 059	16 402	174 489
*Mus musculus*	GRCm38.p4	GRCm38.p4	GRCm38.84	486 198	82 477	30 220	373 501
*Rattus norvegicus*	Rnor_6.0	Rnor_6.0	Rnor_6.0.84	289 077	51 423	6718	230 936
*Bos taurus*	UMD_3.1	UMD_3.1	UMD_3.1.86	220 483	49 026	6942	164 515
*Ovis aries*	Oar_v3.1	Oar_v3.1	Oar_v3.1.89	340 974	40 000	19 022	281 952
*Danio rerio*	GRCz10	GRCz10	GRCz10.84	257 534	56 247	14 523	186 764
*Drosophila melanogaster*	Release 6 plus ISO1 MT	BDGP6	BDGP6.84	97 934	22 204	2148	73 582
*Caenorhabditis elegans*	WBcel235	WBcel235	WBcel235.84	94 087	29 563	2340	62 184
*Saccharomyces cerevisiae S288c*	R64	R64	R64.83	16 865	6613	28	10 224

Ref = currently annotated protein (RefProt), II_ = novel isoforms of known protein, IP_ = novel protein from alternative ORF (AltProt).

### ORF product classification: reference proteins, novel isoforms, and alternative proteins

This exhaustive ORFeome is then filtered using NCBI RefSeq, Ensembl and UniProt (29) protein entries to identify annotated proteins, called RefProts (Figure 1). We add UniProt entries at this level as UniProt contains proteins with supporting experimental evidence that are not present in either NCBI RefSeq or Ensembl annotations. For example in human, 11,860 proteins annotated in UniProt (UniProtKB-SwissProt, 2017-09-27) are not present in either NCBI RefSeq or Ensembl annotations. Once known ORFs are filtered out (RefProt category), we are left with currently unannotated ORFs. A similarity filter is then implemented to identify and annotate Novel RefProt Isoforms (Figure 1). The similarity filter targets ORFs from a same gene and contains two arguments: (a) over 80% of protein sequence identity over 50% of the length (Basic Local Alignment Search Tool (BLAST) (30)), and (b) identical genomic coordinates of start or end codon with a protein sequence identity (EMBOSS Matcher PAM10 matrix score ≤ 100) over 20% of the length (28). If any of the similarity filter argument is met, the unannotated ORF is categorized as a novel predicted isoform (II_ accessions). The leftover predicted ORFs constitute the alternative proteins (AltProt category, IP_ accessions) and can be summarized by a simple equation: AltORFs = ORFeome - RefORFs - Novel Isoforms. All predictions for each species are present on the OpenProt website and can be downloaded, queried or visualized using the Genome Browser. A comprehensive guide for all three is provided under the Help section of the OpenProt website (www.openprot.org/p/help).

### Mass spectrometry data analysis pipeline

In order to gather protein expression evidence, OpenProt retrieves publicly available mass spectrometry (MS) based proteomics studies from ProteomeXchange (31), PRIDE archive (32) and collaborators. Such studies are re-analyzed using the OpenProt protein FASTA containing all RefProts, AltProts and Isoforms. The OpenProt MS pipeline was developed using PeptideShaker software (version 1.13.4) (33) configured to systematically run 4 search engines on raw MS files (X!Tandem, MS-GF+, Comet and OMSSA) via SearchGUI (version 3.1.0) (34). SearchGUI general parameters were set as previously described and then individually inferred based on studies specifics (7). Classical MS analyses use a false discovery rate (FDR) of 1%. However, adding all AltProts and Isoforms leads to a substantial increase of the search space (about six times bigger for human). To only account for highly confident identifications, we set the FDR at 0.001% (Figure 1). Initial validations included: (a) 80% minimum overlap of RefProts identifications with the original MS study, and (b) manual validation of randomly selected spectra (supporting materials S1). Admittedly, false positives may still be and we strongly encourage seeking ORFs with evidence across multiple datasets as false-positive identifications would differ across datasets. Moreover, a novel predicted protein (AltProt or Isoform) will be identified only if it is recognized by a unique peptide. In the case where a peptide matches a novel predicted protein and a RefProt, it will always be assigned to the RefProt only. The identification results are then implemented to the OpenProt database and can be downloaded or queried from the Genome Browser or the Search page. More information can be found on the web platform Help page (www.openprot.org/p/help) and in supporting materials S1.

### Ribosome profiling data analysis pipeline

In order to gather ORF translation evidence, OpenProt retrieves publicly available ribosome profiling (Ribo-seq) data. Ribosomal footprints raw data are re-analyzed using the PRICE workflow (version 1.0.2) (35). PRICE is an entropy-based model for identification of translated ORFs from Ribo-seq data. PRICE is run with default parameters (except for the FDR) using the rescue mode (Figure 1), and fed with both NCBI RefSeq and Ensembl annotations (run separately). Briefly, reads mapping to ribosomal RNAs are filtered out and remaining footprints are mapped onto the genome and transcriptome with up to three mismatches (35). Multi-mapped reads are fractionated across all possible sites unless uniquely mapped reads to nearby loci allow confident identification of the footprint coordinates (35). PRICE reconstitutes the set of codons most likely to give the observed reads, creating a list of ORF candidates. These are filtered according to a stringent 1% FDR (usually set at 10%) to focus on highly confident translation events (35). The identification results are then implemented to the OpenProt database and can be downloaded or queried from the Genome Browser or the Search page. More information can be found on the web platform Help page (www.openprot.org/p/help) and in supporting materials S2.

### Conservation analysis pipeline

In order to gather protein conservation evidence, OpenProt computes orthology relationships from the 10 currently supported species. Protein sequence homology is evaluated using an InParanoid-like approach and separates orthologs (homologous sequences from different species) from paralogs (homologous sequences from the same species but different genes) (36). To identify orthologs, protein sequences from two different species are compared using an all-vs-all BLAST (36). For example, all protein sequences from *Homo sapiens* are BLAST searched against all protein sequences from *Pan troglodytes*. All orthology relationships identified are available on OpenProt (one-to-one; one-to-many; many-to-one and many-to-many). In parallel, the same pipeline is run within one species to identify paralogs (Figure 1). OpenProt uses a significance filter set at a bitscore of 40 for an overlap over 50% of the query sequence, as previously published (7, 37). The results are then implemented to the OpenProt database and can be downloaded or queried from the Search page. More information can be found on the web platform Help page (www.openprot.org/p/help) and in supporting materials S3.

### Protein functional domain prediction

All predicted proteins are also scanned to identify known functional domains. All protein sequences are run through the InterProScan algorithm (version 5.14–53.0) using the default parameters (38). Domain predictions as well as gene ontology (GO) and pathway annotations are reported if significant (e-value < 10^−3^). The results are inserted into the OpenProt database and can be downloaded or queried from the Search page.

### Database and website development

All data are generated using in-house Perl (version 5.18.2) and Python (version 2.7.6) scripts and stored in a PostgreSQL database (version 9.6). All re-analyzed mass spectrometry and ribosome profiling studies are accessible from the Help page (supporting materials S1 and S2). The OpenProt web platform was built using the Flask framework (version 1.0.2) and developed using HTML, SQL and JavaScript.

## DATABASE CONTENT AND USER INTERFACE

### ORFeome: numbers and classification

**Figure 2.**
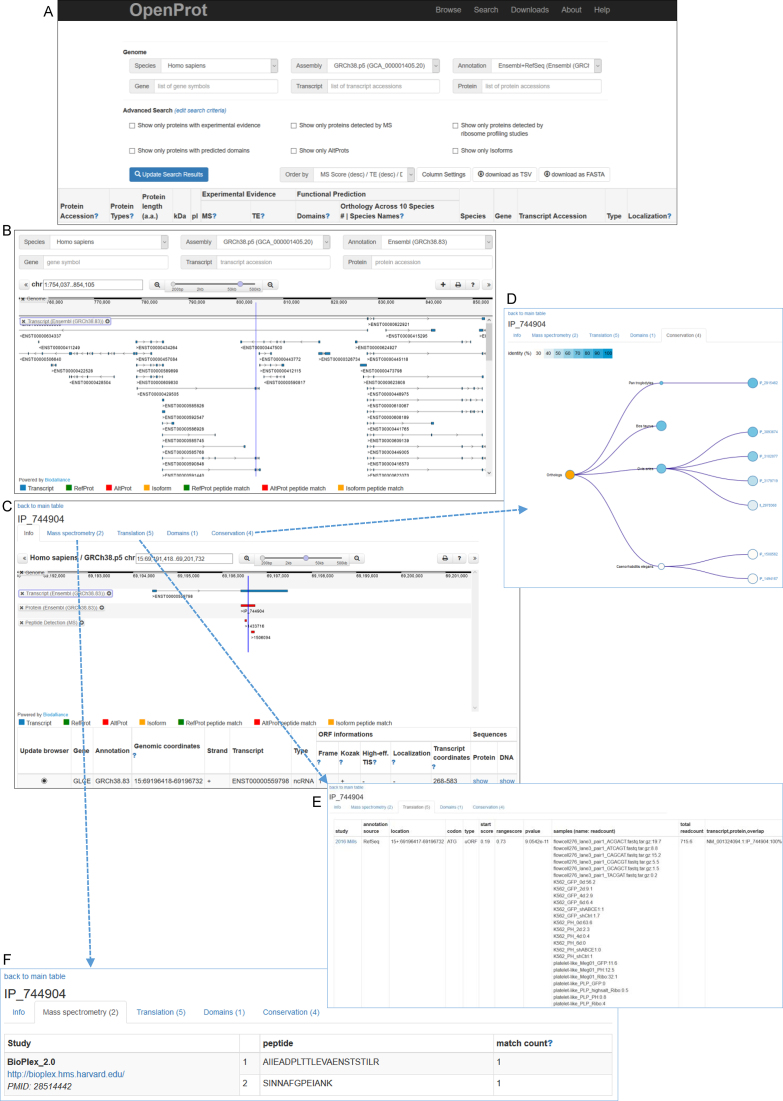
OpenProt features. (**A**) OpenProt Search page. All OpenProt pages are accessible from the top menu. The Search page displays advanced query settings (species, annotation, gene, transcript and protein) and allows to filter according to supporting evidence and other characteristics. The result table displays the main protein characteristics, supporting evidence, and link to a Details page (shown in C). A getting started tutorial from the Search page is available from the help menu (www.openprot.org/p/help). (**B**) OpenProt Genome Browser page. The Browser page displays advanced query settings (species, annotation, gene, transcript, protein and genomic coordinates) and a genome browser with customable tracks. By default, the genome browser shows tracks of transcripts, proteins and peptide detection (from re-analyzed mass spectrometry studies). A getting started tutorial from the Browser page is available from the help menu (www.openprot.org/p/help). (**C**) Each ORF has an individual ‘Details’ page. The page holds 5 tabs, the first one shown here displays information such as genomic and transcript coordinates, and other characteristics. Protein and DNA sequences are available from the details link. (**D**) The ‘Conservation’ tab displays the ORF orthologs and paralogs. The size of the nodes represents the number of related ORFs and the colour shows the identity percentage. The conservation score (top of the tab) corresponds to the number of species in which an ortholog was identified out of the 10 currently supported by OpenProt. (**E**.)The ‘Translation’ tab displays PRICE analysis results with the associated *P*-value and the readcount per sample. The TE score (top of the tab) corresponds to the number of studies in which this ORF was detected. (**F**) The ‘Mass spectrometry’ tab displays the identified peptides with the study name and a link to the original data. The match count corresponds to the number of PSM (peptide spectrum match) per peptide, per study. The MS score (top of the tab) corresponds to the sum of unique peptides per study. More information and tutorials are available from the Help page (www.openprot.org/p/help).

ORF predictions can be queried from either one or both of the annotations or both from the search or browser page (Figure 2A). Numbers of identified RefProts, AltProts and Isoforms are displayed in Table 1 for each species. For example in human to date, OpenProt (release 1.3) predicts 55,053 novel isoforms. These are currently non-annotated in NCBI RefSeq or Ensembl, they originate from transcripts present in current annotations and display significant similarity with the gene annotated CDS (see similarity filter arguments).

All ORF predictions can be visualized using the genome browser where transcripts, ORFs, and MS-based peptides are displayed. For easy visualization, RefProts are coloured in blue, AltProts are in green, and Isoforms are in orange (Figure 2B).

Moreover, all predictions can be downloaded as TSV, BED or FASTA (DNA or Protein) files. These include accession numbers, gene name, nucleotide and protein sequences, and other characteristics. Every downloadable file format is detailed in the attached readme documents.

### Protein characteristics annotation

The result table from a query also contains additional information on the predicted ORF (Figure 2A). The predicted protein characteristics, such as the length (in amino acids), the isoelectric point and molecular weight, are displayed. Each protein annotation is linked to all source transcripts for which accession numbers and type (mRNA or ncRNA) are reported. Localization within the transcript (CDS, 5′ or 3′ UTR) is also displayed for ORF predictions from mRNAs. Each ORF can be inspected individually in the details page (Figure 2C). The details page contains ORF information, such as genomic and transcript coordinates, the presence of a simplified Kozak motif (RNNATGG where R stands for A or G) (39) or a high-efficiency translation initiation motif (RYMRMVAUGGC where R stands for A or G, Y for U or C, M for A or C, and V for A, C or G) (40) and access to protein and DNA sequences. Other tabs display specifics of lines of evidence: protein conservation, MS, translation events and protein domain prediction.

### Supporting evidence annotation

OpenProt first predicts potential ORFs and then collects diverse types of evidence, such as functional predictions (protein conservation and predicted domains) and experimental evidence (translation event and protein expression detection). Numbers of predicted ORFs supported by conservation, translation or expression evidence are displayed in Table 2 per ORF category for each species.

**Table 2.** OpenProt (1.3) evidence collection output

	Conservation evidence				Translation evidence (Ribo-seq)				Protein evidence (MS)			
Species	Sp #	Ref #	II_ #	IP_ #	St #	Ref #	II_ #	IP_ #	St #	Ref #	II_ #	IP_ #
*Homo sapiens*	9	189 319	38 325	239 394	33	17 435	2048	5 696	62	113 006	1455	28 641
*Pan troglodytes*	9	63 408	28 930	148 989	0	*n/a*	*n/a*	*n/a*	0	*n/a*	*n/a*	*n/a*
*Mus musculus*	9	131 130	21 245	121 890	22	14 607	1 088	3081	28	61 440	165	2 877
*Rattus norvegicus*	9	82 951	5354	81 600	2	6661	202	870	8	21 282	19	410
*Bos taurus*	9	70 697	6086	88 550	0	*n/a*	*n/a*	*n/a*	1	12 778	5	37
*Ovis aries*	9	56 331	28 247	107 900	0	*n/a*	*n/a*	*n/a*	1	1 466	18	69
*Danio rerio*	9	81 958	19 560	8 965	2	9	1	0	7	26 114	263	386
*Drosophila melanogaster*	9	39 246	763	452	3	2453	39	113	3	9783	20	113
*Caenorhabditis elegans*	9	28 429	861	450	5	8142	161	84	0	*n/a*	*n/a*	*n/a*
*Saccharomyces cerevisiae S288c*	9	5842	5	38	20	5357	4	283	4	4028	0	20

Sp = Number of species evaluated for orthology relationships (not counting the queried species); St = number of studies re-analyzed by OpenProt; Ref = currently annotated CDS (RefORF); II_ = novel isoforms of known CDS; IP_ = novel CDS from alternative ORF (AltORF); n/a = when no dataset has been re-analysed for this species yet (OpenProt release 1.3). Conservation evidence = all proteins with at least one ortholog in at least one species. Translation evidence = all ORFs detect in at least one detection by PRICE analysis of Ribo-seq data. Protein evidence = all proteins with at least one unique peptide in at least one study.

Protein orthology and paralogy relationships can be visualized for each protein annotated in OpenProt (Figure 2D). Under the Conservation tab of each protein Details page, orthologs and paralogs are listed per species. Similarly, predicted functional domains, gene ontology and pathway terms can be browsed under the Domains tab (Details page).

Experimental evidence is accessible under the Translation and Mass spectrometry tabs (Details page). The Translation tab collects PRICE (35) reports of identifications with the associated *P*-value and read count per sample (Figure 2E). The Mass spectrometry tab contains all identified unique peptides alongside the associated peptide spectrum match (PSM) count within each dataset (Figure 2F). For transparency purposes, all original datasets are accessible by clicking on the study name. At this time, the database re-analyzed 87 ribosome profiling and 114 mass spectrometry studies across several species and diverse cell lines and tissues (Table 2). A complete list of all studies implemented in OpenProt is available from the Help section (supplementary materials S1 and S2).

### Applications and downloads

Expanded databases, such as OpenProt, are invaluable tools for functional proteomics discoveries (2,4,6,7). OpenProt is tailored for every need of all researchers, giving them the required tools for a more comprehensive view of eukaryotic genomes’ coding potential. That is why in addition to the genome Browser and the advanced query page, the Downloads page allows users to download custom databases. For example, one may choose to focus on only the most confident annotations (previously unannotated proteins detected with at least two unique peptides in mass spectrometry experiments), when another may want to focus on discovery of novel functional proteins (all predictions). Moreover, personalized database generation and download based on custom RNA-seq results is also supported, as detailed under the Help section (supplementary materials S1). The results table from specific queries can also be shared or downloaded as a TSV file or as a protein FASTA file. Overall, OpenProt allows (1) advanced search and download of results table, (2) genome browsing with visualization of MS evidence, and (3) personalized downloads (data and file format) for any endeavour.

## DISCUSSION AND COMPARISON TO EXISTING RESOURCES

OpenProt annotates thousands of novel predicted proteins supported by experimental evidence and functional predictions. As more Ribo-seq and MS datasets are constantly added to the database, we expect this number to rise. The out-of-focus resolution at which we currently look at eukaryotic coding potential and proteomes is gradually being acknowledged (1,3,5,18,20), yet we still lack systematic approaches to the problem and this could impede on our understanding of basic biology questions (6).

To the best of our knowledge, OpenProt is the first database that fully endorses a polycistronic model of mammalian genome annotation. OpenProt differs from other smORFs databases in that it does not uphold a maximum length threshold (below 100 codons for smORFs). OpenProt also allows for multiple ORFs per transcript, and supports two transcriptome annotations. Furthermore, the OpenProt pipeline allows for the identification and detection of novel isoforms. Thus, OpenProt reaches a deeper ORF annotation throughout the genome.

In addition, OpenProt distinguishes itself from UniProt as it provides a graphical interface that allows the user to browse all predicted ORFs, in addition to providing functional annotations (conservation, translation, expression and presence of functional domains). OpenProt does not currently allow for deep functional annotation, such as UniProt does. However, when a novel protein is discovered and sufficiently characterized to meet UniProt annotation requirements, it will then become a RefProt in OpenProt database (with a UniProt accession). Thus, OpenProt is a modern tool that fills a major gap in the field of functional annotation of proteins by fostering less serendipitous discoveries of novel proteins.

## FUTURE DIRECTIONS

The OpenProt pipeline is automated so that new releases of NCBI RefSeq and/or Ensembl will automatically be taken into account. Following iGenomes update, data will be updated at the beginning of the following year for computational resources access reasons. OpenProt is a release-based platform, developed in accordance to the FAIR guiding principles for scientific data management and stewardship (41). This ensures an up-to-date, continuous availability of all OpenProt data through time.

As the field progresses, OpenProt is expected to elaborate its pipeline, notably taking in account ORFs shorter than 30 codons or non-ATG start codons. For statistical and computational reasons, OpenProt current release (1.3) still holds these cut-offs. Simultaneously, OpenProt will continue to increase the number of datasets for supporting evidence, as well as supported species. OpenProt can be contacted through the Contact page for request of species annotations and/or dataset analyses (http://www.openprot.org/p/about). New tools and metrics will also be added following developments in the field. For instance, a pipeline is being developed to allow AltORFs and novel Isoforms consideration in genome or exome sequencing studies.

The quantity and quality of data provided by OpenProt along with its ease of use and transparent data availability hold potential to make it a popular tool.

## Supplementary Material

Supplementary Data
