# Supplementary material for: OpenProt: a more comprehensive guide to explore eukaryotic coding potential and proteomes
Source: Nucleic Acids Res. 2018 Oct 9;47(Database issue):D403–10. doi: 10.1093/nar/gky936 (PMC6323990; doi:10.1093/nar/gky936)
Supplement: Supplementary Data [file gky936_supplemental_files.pdf]

Supporting Materials S1

I. MS Spectra validation examples

(1) Novel Isoform with high MS score: **II_794710**

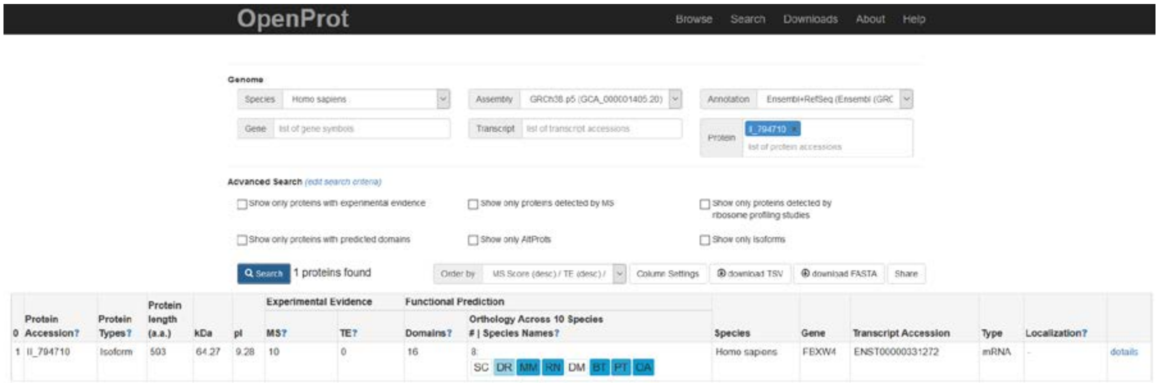

From PeptideShaker, peptide from TCGA_BRCA study (*PMID: 27251275*).

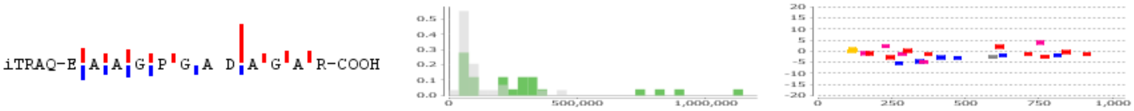

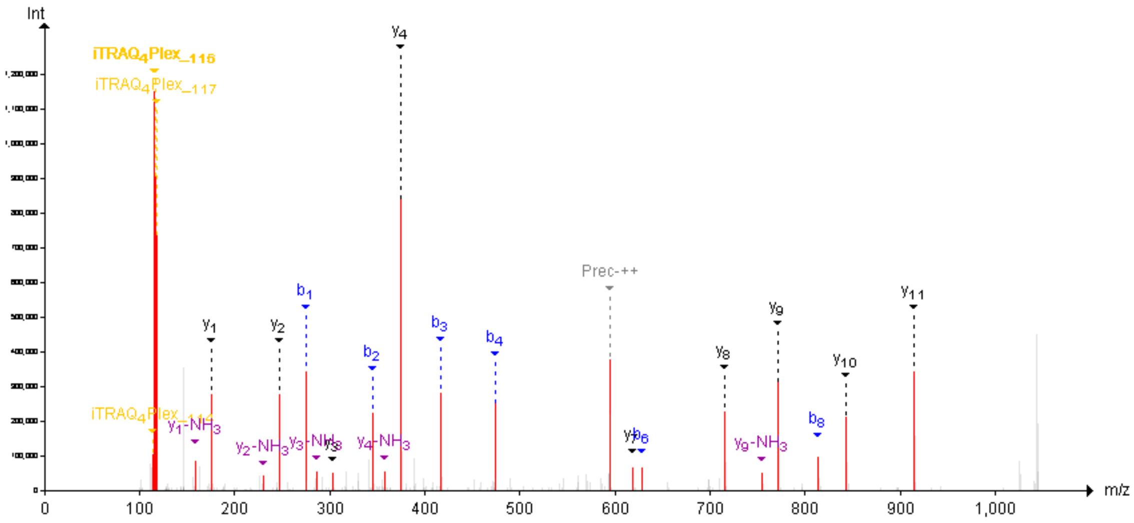

(2) Novel Isoform with low MS score: **II_683445**

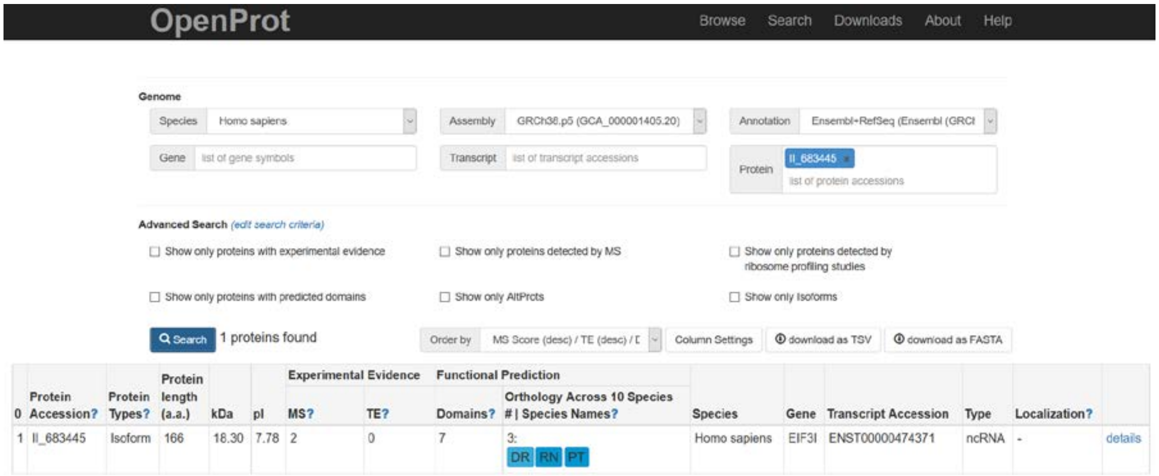

0	Protein Accession?	Protein Types?	Protein length (a.a.)	kDa	pI	Experimental Evidence MS?	TE?	Functional Prediction Domains?	Orthology Across 10 Species # | Species Names?	Species	Gene	Transcript Accession	Type	Localization?	
1	II_683445	Isoform	166	18.30	7.78	2	0	7	3: DR RN PT	Homo sapiens	EIF3I	ENST00000474371	ncRNA	-	details

From PeptideShaker, peptide from Bioplex study (*PMID: 28514442*).

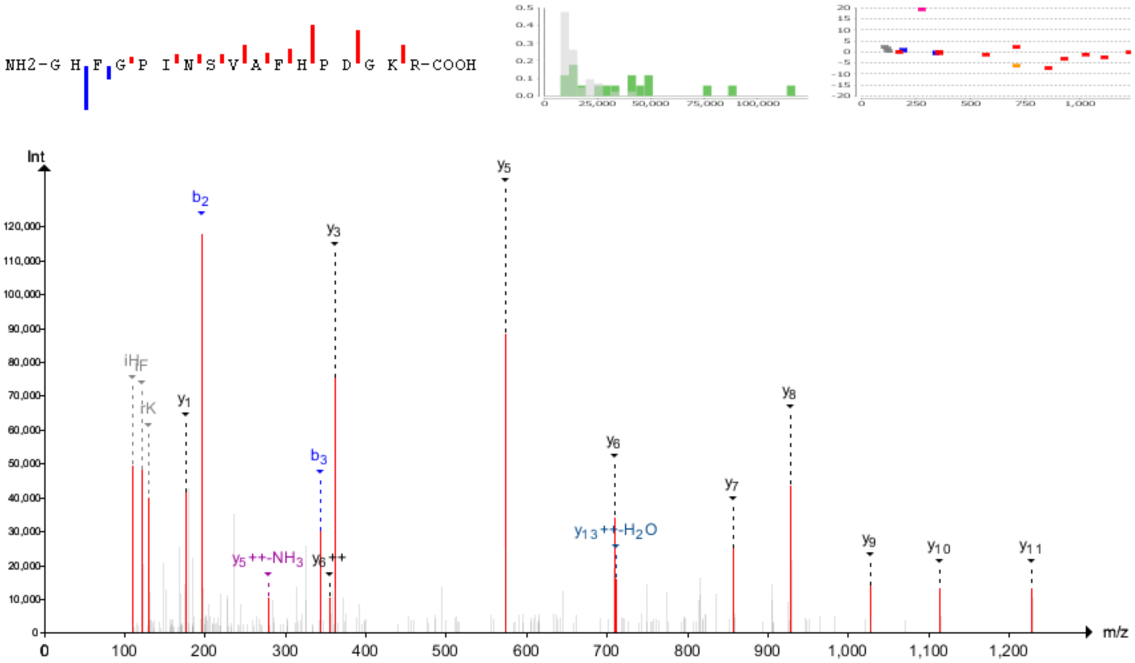

(3) Alternative Protein with high MS score: **IP_079312**

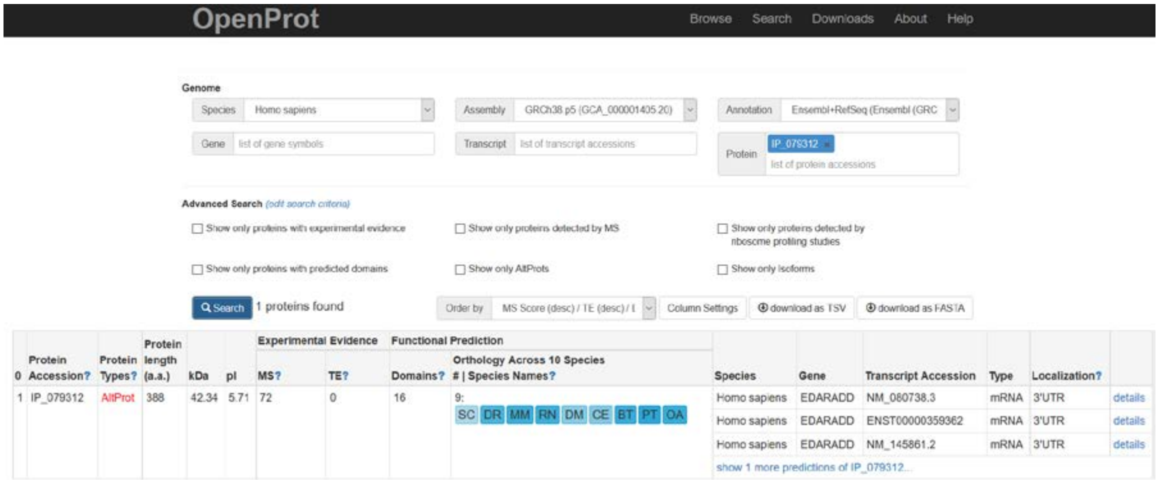

From PeptideShaker, peptide from Bioplex study (*PMID: 26186194*).

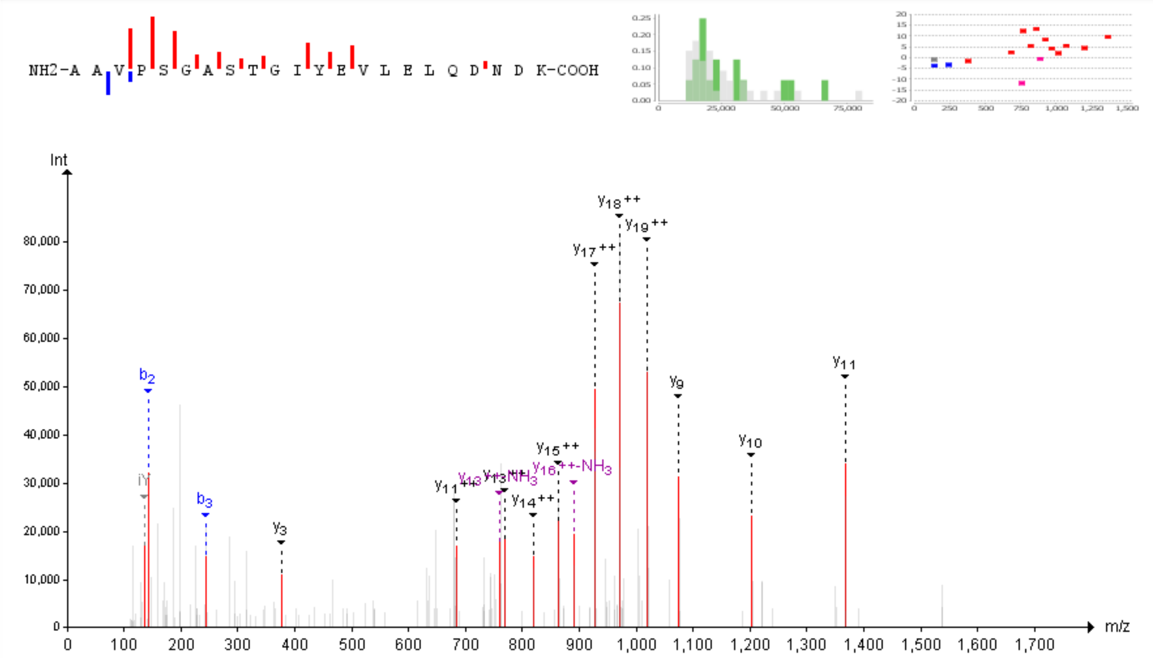

(4) Alternative Protein with low MS score: **IP_130992**

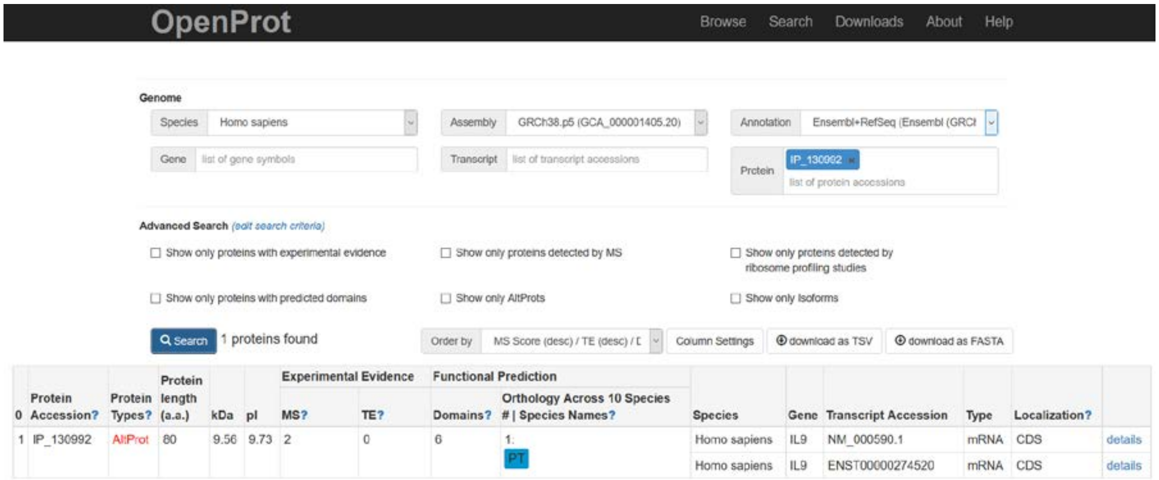

From PeptideShaker, peptide from TCGA_BRCA study (*PMID: 27251275*).

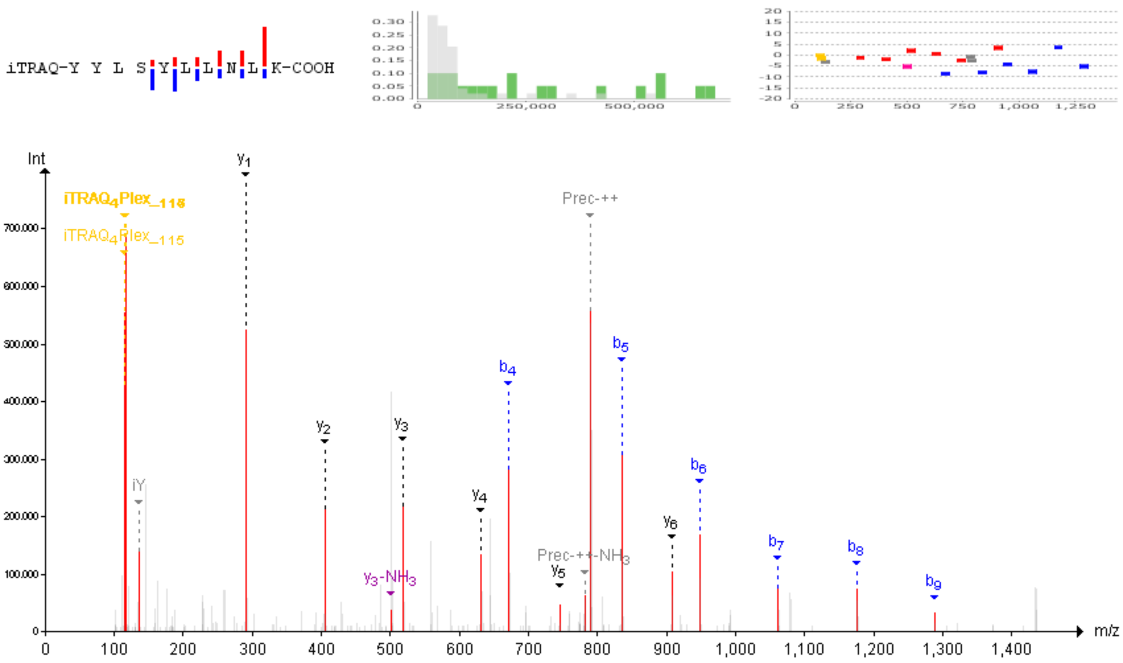

II. MS pipeline additional information

Once downloaded from ProteomeXchange [31], PRIDE archive [32] and collaborators, original RAW files are converted to mzXML. This conversion is done using ProteoWizard (version 3.0.8789). The converted files can then be analysed on the mp2 supercomputer (Calcul Québec, Calcul Canada) in a linux environment (CentOS 6.8). This computer of 1632 nodes of 24 cores each allows rapid re-analysis of large datasets.

III. Help Materials for mass spectrometry identifications

- How is the MS score calculated?
- How does OpenProt identify AltProts in MS-based proteomic analyses?
- How is the increase in the database search space accounted for?
- How does OpenProt deal with comparability and reliability across MS datasets?
- I have some MS datasets I would like to re-analyze using OpenProt, what should I do?
- I have MS datasets that I would like to share with OpenProt, how can I do so?
- I have an RNA-seq dataset and would like to download a custom fasta with OpenProt, how can I do so?
- Where can I find a list of MS experiments re-analyzed by OpenProt?
- Where can I find a link to a specific study my protein of interest was detected in?
- Peptide assignation across isoforms: what are the rules?
- Can proteins coded by RNAs currently annotated as non-coding (pseudogenes RNAs and lncRNAs) be detected?
- MS detection truths: recommendations

How is the MS score calculated?

The Mass spectrometry score (MS score) is the **sum of unique peptides identified** per MS-studies reanalyzed by OpenProt.
In order to be annotated as detected by MS, several criteria have to be met:

(1) Only unassigned peptides from MS data can be matched to AltProts. If a peptide matches both a RefProt and an AltProt, the corresponding Peptide Spectrum Match (PSM) will be assigned to the RefProt only. *For more information on the MS annotation pipeline enforced by OpenProt, see below. For more information on peptide assignation rules, click here.*

(2) An AltProt or Isoform must have been seen with at least one unique peptide.

As such, if an AltProt is detected by one unique peptide in three different MS datasets, and two different unique peptides in one other MS study, the corresponding MS score is 5 (1+1+1+2).

Back

How does OpenProt identify AltProts in MS-based proteomic analyses?

OpenProt retrieves MS raw data files from ProteomeXchange [31], PRIDE archive [32] and collaborators. Each MS data file is then analysed using the same pipeline, a stringent **FDR of 0.001%** is enforced. Traditionally, a 1% FDR is used; we chose a more stringent FDR to focus on high quality AltProt identifications only. The decoy database contains the reversed sequences of proteins from the target database. All peptide sequences identified for a protein (RefProt, Isoform or AltProt) can be seen under the **details** tab.

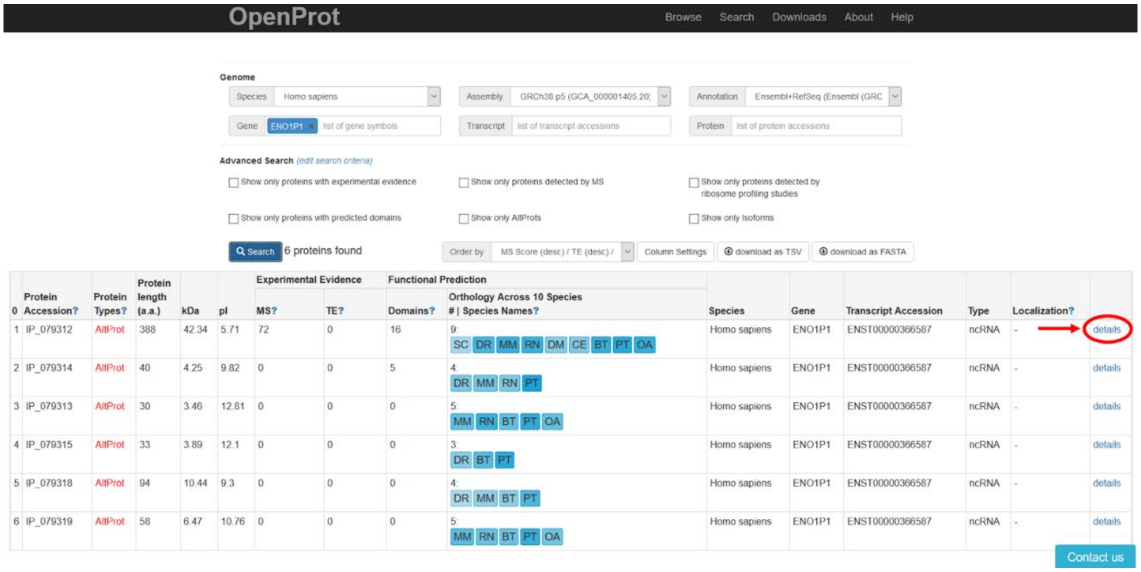

Then you can click on the **Mass Spectrometry** tab to display details about evidence of expression from MS datasets.

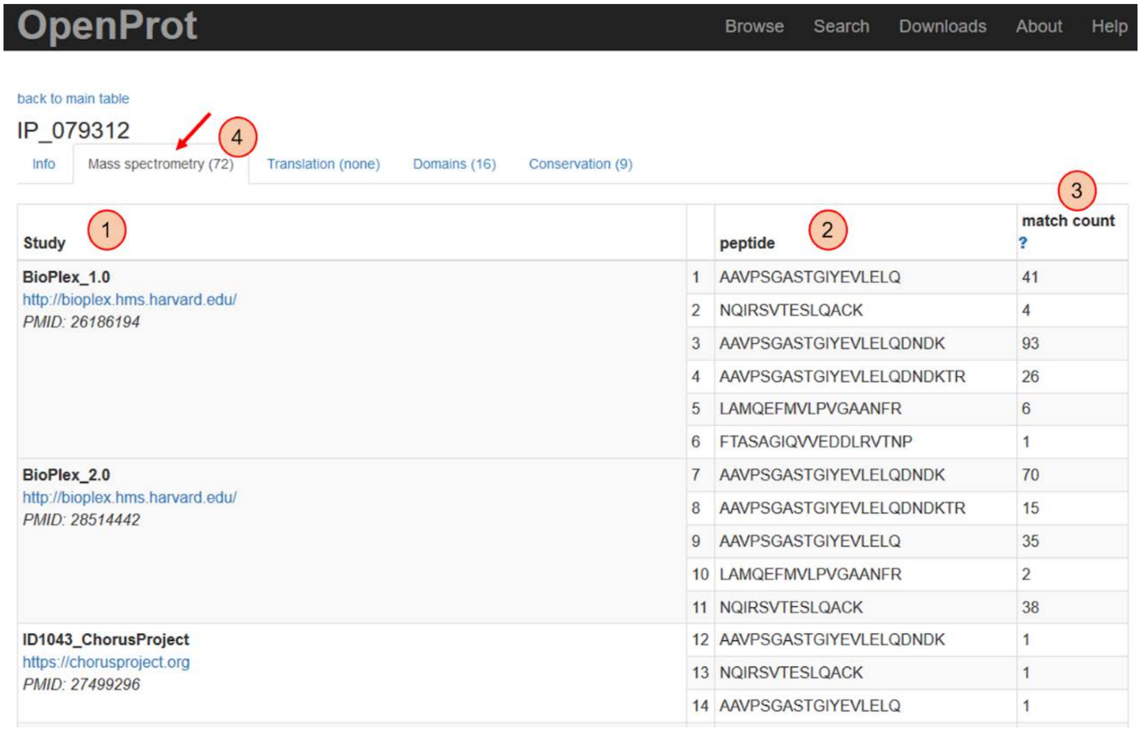

Study		peptide	match count ?
BioPlex_1.0<br>http://bioplex.hms.harvard.edu/<br>*PMID: 26186194*	1	AAVPSGASTGIYEVLELQ	41
	2	NQIRSVTESLQACK	4
	3	AAVPSGASTGIYEVLELQDNDK	93
	4	AAVPSGASTGIYEVLELQDNDKTR	26
	5	LAMQEFMVLPVGAANFR	6
	6	FTASAGIQVVEDDLRVTNP	1
BioPlex_2.0<br>http://bioplex.hms.harvard.edu/<br>*PMID: 28514442*	7	AAVPSGASTGIYEVLELQDNDK	70
	8	AAVPSGASTGIYEVLELQDNDKTR	15
	9	AAVPSGASTGIYEVLELQ	35
	10	LAMQEFMVLPVGAANFR	2
	11	NQIRSVTESLQACK	38
ID1043_ChorusProject<br>https://chorusproject.org<br>*PMID: 27499296*	12	AAVPSGASTGIYEVLELQDNDK	1
	13	NQIRSVTESLQACK	1
	14	AAVPSGASTGIYEVLELQ	1

In the first column, you will find the **name of the study** analysed (**1**), along with a link and the reference. For each study, you will find in the second column the list of **identified peptides** (**2**), and the third column indicates the number of **peptide spectrum matches** (**3**). Finally, next to the tab title (**4**), the number between brackets refers to the overall **MS score**, as explained here.

Back

How is the increase in the database search space accounted for?

Adding all predicted AltProts and novel Isoforms to the database corresponds to an additional 516,515 entries in Human alone, leading to a substantial increase in the search space. Thus, OpenProt enforces a **stringent FDR of 0.001 %** (traditionally, set at 1 %), in order to focus on highly confident AltProts and Novel Isoforms identifications, and to account for the database increased size. This strategy was designed with the kind help of Peptide Shaker developers, initial validations included: (a) a minimum of 80% overlap of RefProts identifications with the original MS study; and (b) a manual validation of randomly selected spectra.

Back

How does OpenProt deal with comparability and reliability across MS datasets?

OpenProt pipeline retrieves publicly available top-down MS/MS datasets mostly from PRIDE and ProteomeXchange. That ensures datasets have been run through the PRIDE Inspector to assess data quality (PMID 22318026) and that they follow the ProteomeXchange consortium guidelines (PMID 27924013). Moreover, for each retrieved dataset, parameters are validated through text-

mining and manual curation. As such, OpenProt ensures to analyze data from high quality mass spectrometers and to do so with the appropriate parameters. Although this step is time consuming, it guarantees higher quality data and reliability on OpenProt.

Back

I have some MS datasets I would like to re-analyze using OpenProt, what should I do?

All data on OpenProt is freely available and downloadable. You can thus download custom fasta files to analyze your MS datasets. *For more information on downloads, click here. For more information on which database to download, click here.*

Back

I have MS datasets that I would like to share with OpenProt, how can I do so?

We are constantly adding datasets to the OpenProt database. If you have some you would be willing to share with us, you can contact us here.

Back

I have an RNA-seq dataset and would like to download a custom fasta with OpenProt, how can I do so?

OpenProt allows the download of custom database to couple MS studies with RNA-seq experiments for example. Under the search page, you enter your list of transcripts (**1**) - or your list of genes (**1**) - and click on search (**2**). This will display your table of results, and you can then download these as a fasta file (**3**).

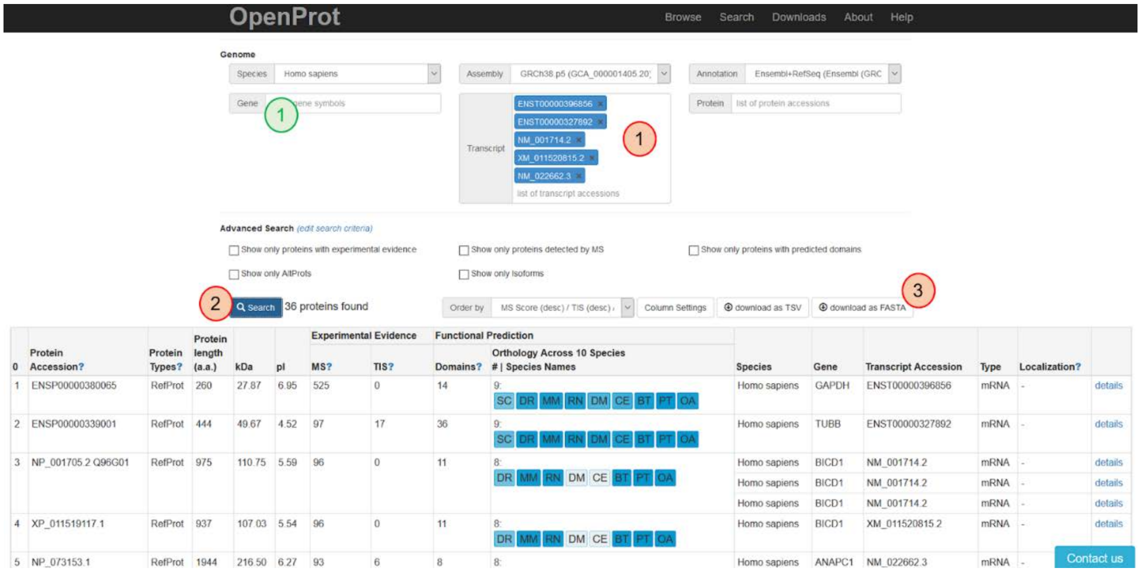

For more information on the fasta header, click here.

Nota bene: For the moment, there is a limit at 2,000 gene / transcript entries at a time. However, you can download it as several fasta files and then concatenate them; or download all sequences in the Downloads section and filter by your genes / transcripts of interest.

Back

Where can I find a list of MS experiments re-analyzed by OpenProt?

A full list of mass spectrometry studies added to the OpenProt database can be found here.

Back

Where can I find a link to a specific study my protein of interest was detected in?

Under the Details page of a specific protein, you can click on the Mass Spectrometry tab (**1**). The first column (**2**) contains the details about each specific study. It notably contains a link to the data (**3**) and the Pubmed ID to the related publication (**4**).

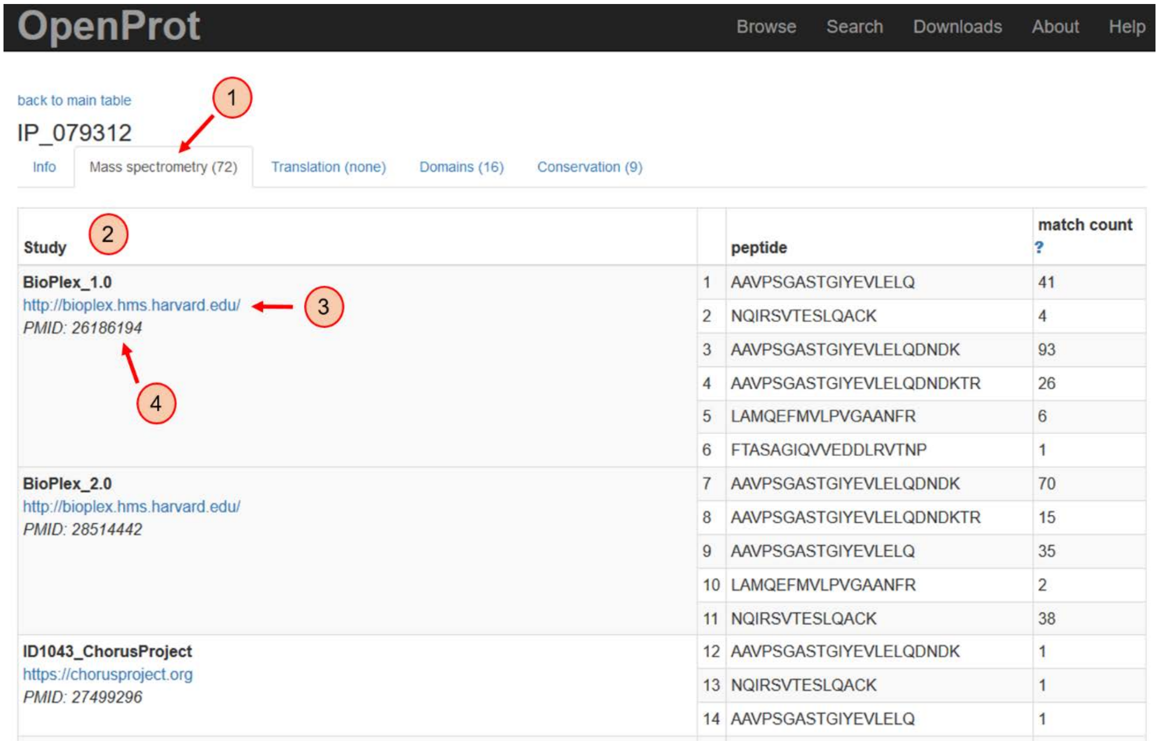
OpenProt Browse Search Downloads About Help

back to main table

IP_079312

Info | Mass spectrometry (72) | Translation (none) | Domains (16) | Conservation (9)

Study		peptide	match count ?
BioPlex_1.0 http://bioplex.hms.harvard.edu/ *PMID: 26186194*	1	AAVPSGASTGIYEVLELQ	41
	2	NQIRSVTESLQACK	4
	3	AAVPSGASTGIYEVLELQDNDK	93
	4	AAVPSGASTGIYEVLELQDNDKTR	26
	5	LAMQEFMVLPVGAANFR	6
	6	FTASAGIQVVEDDLRVTNP	1
BioPlex_2.0 http://bioplex.hms.harvard.edu/ *PMID: 28514442*	7	AAVPSGASTGIYEVLELQDNDK	70
	8	AAVPSGASTGIYEVLELQDNDKTR	15
	9	AAVPSGASTGIYEVLELQ	35
	10	LAMQEFMVLPVGAANFR	2
	11	NQIRSVTESLQACK	38
ID1043_ChorusProject https://chorusproject.org *PMID: 27499296*	12	AAVPSGASTGIYEVLELQDNDK	1
	13	NQIRSVTESLQACK	1
	14	AAVPSGASTGIYEVLELQ	1

Back

Peptide assignation across isoforms: what are the rules?

In the case of two possible assignations on **different genes**, the peptide is **unassigned**.
In the case of two possible assignations **from the same gene**, the rules are detailed below for every encountered combination:

a. If a **RefProt** is amongst the possible assignations, the peptide will **always be assigned to the RefProt**.
b. Assignation possible to **two RefProts**, the peptide is assigned to **both**
c. Assignation possible to **two Novel Isoforms** (II_), the peptide is assigned to **both**.
d. Assignation possible to **two AltProts** (IP_), the peptide is assigned to **both**.
e. Assignation possible to a **Novel Isoform** (II_) and an **AltProt** (IP_), the peptide is assigned to **both**.

Therefore, when a Novel Isoform (II_) or AltProt (IP_) is detected by MS, it is necessarily with a specific peptide that doesn't match the associated RefProt or any other RefProt. **Nota bene**: rules (a) to (e) only apply when assignations refer to different proteins **from the same gene**. If it is a different gene, the peptide is unassigned.

Back

Can proteins coded by RNAs currently annotated as non-coding (pseudogenes RNAs and lncRNAs) be detected?

OpenProt annotates all ORFs (starting with an ATG and longer than 30 codons) and the corresponding AltProts in coding and non-coding RNAs. If non-coding derived AltProts have characteristics that allow detection by MS, then they can be detected. *For more information on MS detection for novel proteins, click here.*

Back

MS detection truths: recommendations

One has to always remember that a vast majority of MS datasets re-analyzed by OpenProt uses a trypsin digestion. Thus, **some proteins may not be detectable** under these conditions. An absence of detection does not mean the protein does not exist.

Moreover, AltProts are mostly **small proteins** (median length of 45 amino acids) which decrease the likelihood of detectable, sufficiently long, unique peptides. Furthermore, some protocols may be in favor of large and abundant proteins, when small proteins detection might require specific protocols (see Ma *et al*., Anal Chem, 2016).

Finally, Novel Isoforms or AltProts might not be identifiable using our OpenProt pipeline if no tryptic peptides permits a **unique assignation**. Indeed, when a peptide can also be assigned to a RefProt, it will always be assigned to the RefProt (see peptide assignation rules).

Back

SUPPORTING MATERIALS S2

Help Materials for translation events (TE) identification

- What does TE and TIS mean?

- What is the TE score?

- What does PRICE do?

- How are translation event detection displayed on OpenProt?

- How should I interpret the p-value?

- How are multi-mapped reads accounted for?

- Where can I find a list of ribosome profiling studies analyzed by OpenProt?

- Where can I find a link to a specific study my protein of interest was detected in?

- Can the translation of pseudogenes be detected?

- I have ribosome profiling datasets that I would like to share with OpenProt, how can I do so?

- TE (Translation Events) detection truths: recommendations

What does TE and TIS mean?

TE stands for Translation Event, when TIS stands for Translation Initiation Sites.

Back

What is the TE score?

The TE score displayed on the search results page and on the Translation tab corresponds to the **number of studies in which a significant identification was made**. *For more information on translation events identification, see below.*

Back

What does PRICE do?

PRICE is an entropy based model for **identification of translated Open Reading Frames** (ORFs) from ribosome profiling datasets. It stands for **PR**obabilistic **I**nference of **C**odon activities by an **E**M algorithm (see PMID 29529017). PRICE uses parameters inferred from well-translated, annotated ORFs to model the stochastic events in ribosome profiling. In brief, a given codon in a

ribosomal P site can produce several footprints, PRICE uses Maximum Likelihood algorithms to reconstitute the set of codons more likely to give the observed reads. The set of codons are then assembled in ORF candidates, where a machine-learning algorithm predicts the start codon. Detected ORFs are then filtered according to a stringent **FDR of 1%** (traditionally set at 10%) to focus on highly confident translation event. *For more information on the PRICE algorithm, see PMID 29529017.*

Back

How are translation event detection displayed on OpenProt?

PRICE results are crossed against OpenProt database. Results summary can be visualized in the TE (Translation Event) column from the main results table (**1**). The detailed results can be seen by clicking on the **details** tab.

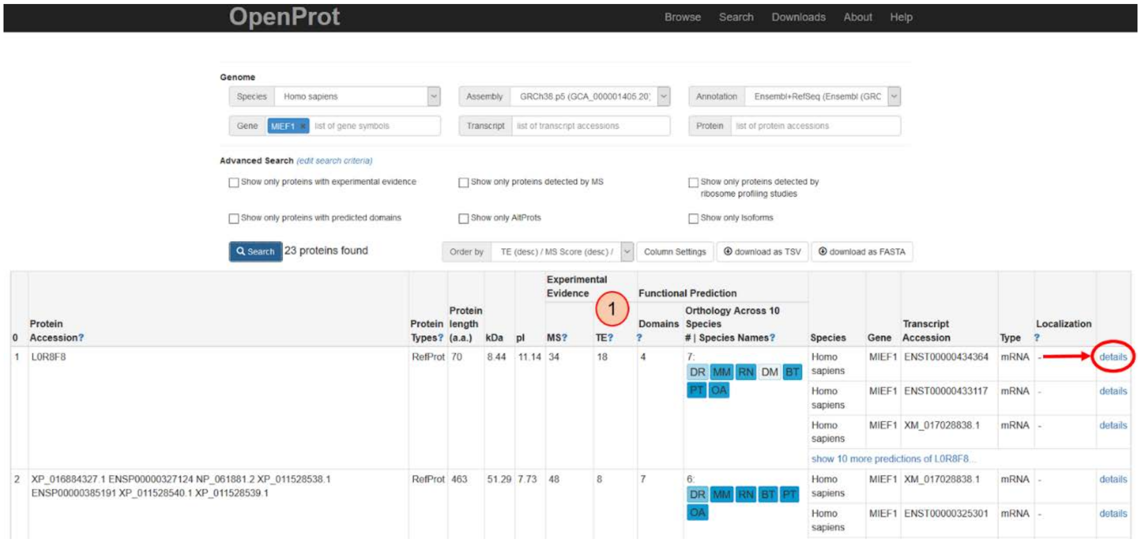

From the details page, you can click on the **Translation** evidence tab.

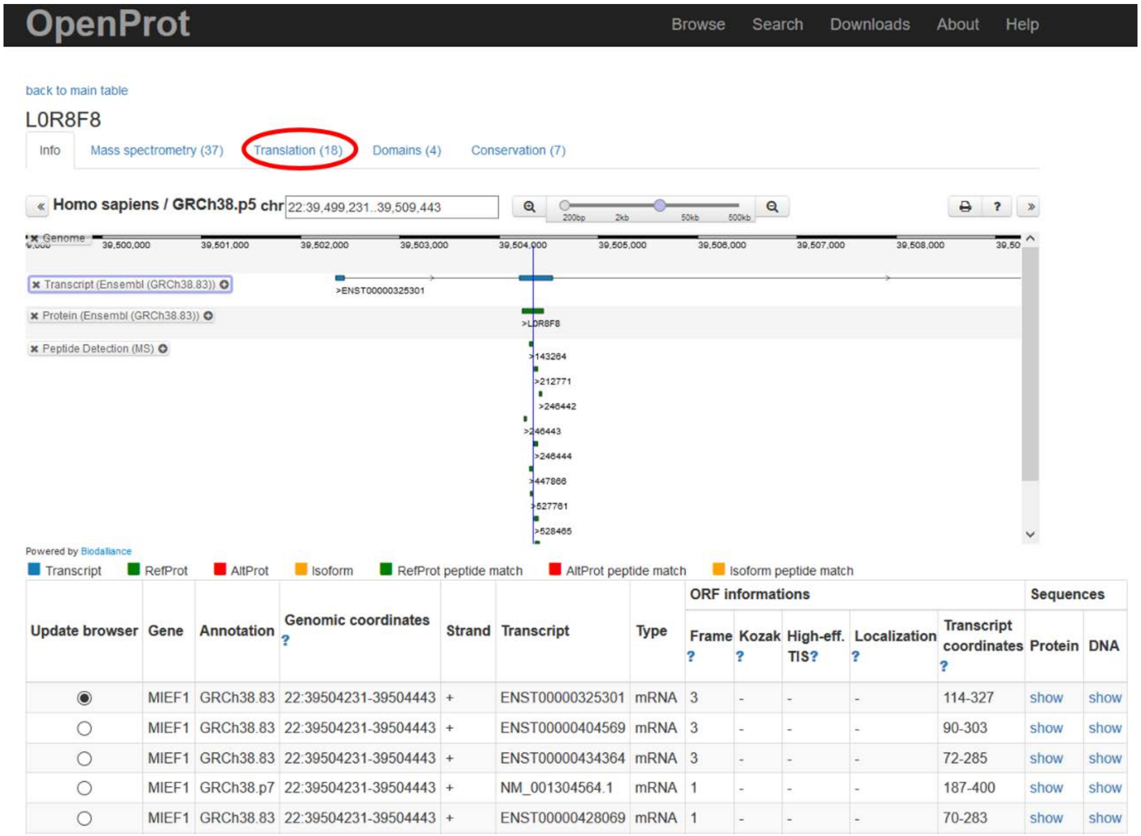

Under the Translation tab, you will find a table with all the studies in which this ORF has been detected (**1**). The detections will be separated based on the annotations (RefSeq, **1**; or Ensembl, **2**).

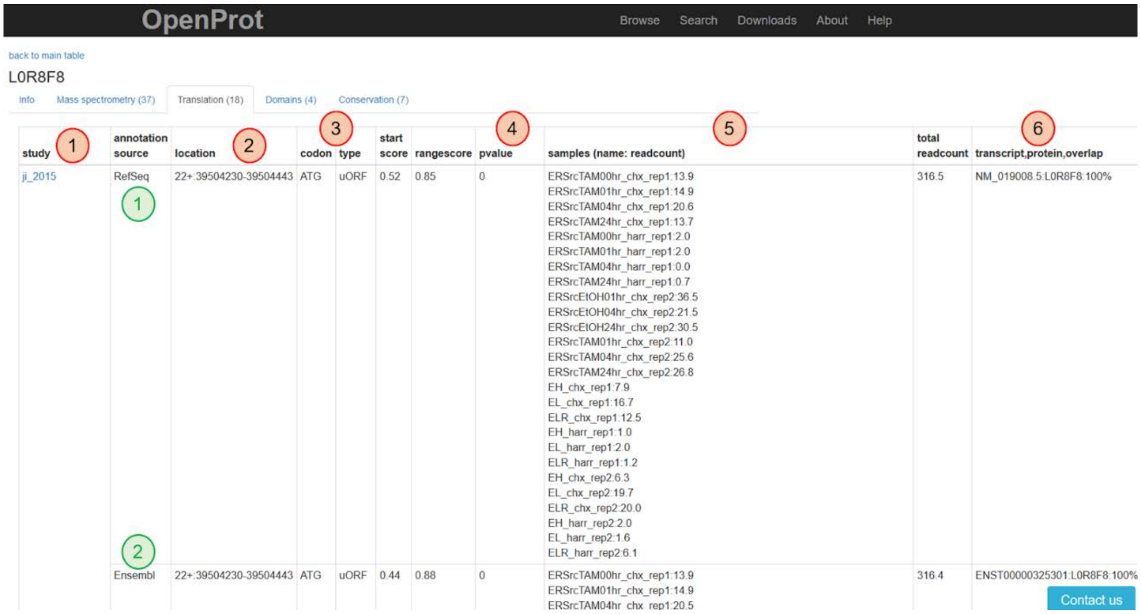

The third column corresponds to the genomic coordinates of the detected ORF (**2**), followed by the start codon and [type of ORF](#) (**3**). The p-value corresponds to the ORF identification confidence

(**4**, *for more information on the p-value, click here*). The samples column (**5**) lists all samples within an experiment with the associated readcount for this ORF. The last column (**6**) corresponds to the transcript and associated protein accessions followed by the overlap of the PRICE predicted ORF sequence with the OpenProt predicted ORF sequence.

Back

How should I interpret the p-value?

The p-value associated to an ORF detection corresponds to the significance of a generalized binomial test (not corrected for multiple comparisons). In brief, it indicates the **confidence of that ORF not being attributable to noise**. In ribosome profiling experiments, noise can arise from (1) ribosomal scanning, (2) abortive translation events in the leader region, (3) non-ribosome mediated mRNA protection from RNAses, or (4) overlapping ORFs. **Nota bene**: this p-value indicate the confidence of the ORF identification, not the confidence of its detection which would be represented by the enforced 1% FDR (see above).

Back

How are multi-mapped reads accounted for?

We run PRICE using the "rescue" mode. This means that if a footprint maps at several places in the genome, the read is either **fractionated or rescued** if uniquely mapped reads nearby allow confident identification of the footprint genomic coordinates.

Back

Where can I find a list of ribosome profiling studies analyzed by OpenProt?

A list of ribosome profiling studies analyzed by OpenProt can be found here.

Back

Where can I find a link to a specific study my protein of interest was detected in?

Under the Details page of a specific protein, you can click on the Translation tab (**1**). The first column (**2**) contains the details about each specific study. The name of the study (**3**) is a link to the original study.

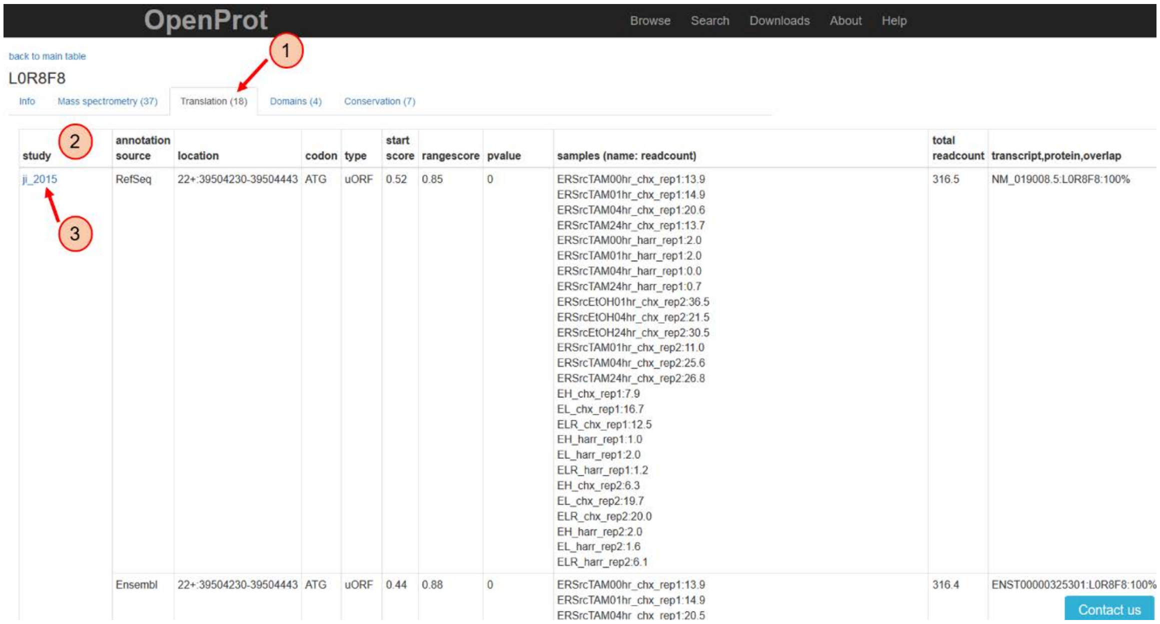

[Back](#)

Can the translation of pseudogenes be detected?

Pseudogenes are by definition an imperfect copy of a functional gene. Thus, pseudogenes share a **high degree of homology with their related genes** and this may hinder their detection by ribosome profiling. Indeed, most of the footprints will multimap to the gene and the pseudogene since footprints are short fragments. OpenProt enforces a pipeline that may hinder pseudogene detection but that favour highly confident annotations. Thus, **a pseudogene may be detected with our pipeline only if unique footprints can be seen**.

[Back](#)

I have ribosome profiling datasets that I would like to share with OpenProt, how can I do so?

We are constantly adding datasets to the OpenProt database. If you have some you would be willing to share with us, you can contact us [here](#).

[Back](#)

TE (Translation Events) detection truths: recommendations

The OpenProt pipeline for ribosome profiling dataset analyses uses [PRICE algorithm](#). It is a model, and thus it may not always fully converge and use the same parameters. Therefore, we **encourage seeking detections across multiple datasets**. Similarly to mass spectrometry data, the more an ORF would have been identified in ribosome profiling datasets, the more confident we are.
In an effort to focus on highly confident translation event, we use a stringent 1 % FDR and a pipeline that may hinder detection of pseudogenes (see [above](#)). Furthermore, identifications are dependent on the quality of the study analyzed (signal to noise ratio, and sequencing depth). Some transcripts may not be seen at all in an experiment. Thus, it is important to remember that **an absence of detection does not mean an absence of translation**.

[Back](#)

SUPPORTING MATERIALS S3

Help Materials for protein orthology analysis

- What is an ortholog and a paralog?
- What is the InParanoid approach?
- How can I see if a protein is conserved?
- I found a novel protein but it is weakly conserved, is it a random ORF?
- Conservation analysis truths: recommendations

What is an ortholog and a paralog?

An **ortholog** is a protein sequence from a species that shares a high degree of homology with a protein sequence from another species. Two orthologous proteins are 2 **similar proteins** from **different species**. Thus, orthologs have a common ancestor gene and diverged by a **speciation** event.
A **paralog** is a protein sequence from a species that shares a high degree of homology with a protein sequence from a different gene within the same species. Two paralogous proteins are 2 **similar proteins** from **different genes** within **one species**. Thus, paralogs originate from a **duplication** event, creating a "copy" of an existing gene [36].

Back

What is the InParanoid approach?

The InParanoid algorithm (PMID 25429972) aims to identify ortholog and paralog groups. The algorithm consists of a all-vs-all Basic Local Alignment Search Tool (BLAST) comparison of all protein sequences in two species. For example, all proteins from *Homo sapiens* are BLAST searched against all proteins from *Pan troglodytes*. Several type of orthologies can be identified, all included in OpenProt : one-to-one corresponds to a pairwise best reciprocal hit (BRH); one-to-many corresponds to all orthologs to one query protein; many-to-one corresponds to all queries matching to one ortholog; and many-to-many corresponds to all orthologs to all queries. Secondly, the same can be done within one species to identify paralogs. OpenProt uses a significance filter at a bitscore of 40 for an overlap over 50 % of the query sequence, as previously published (Samandi *et al.*, eLife, 2017). *For more information on InParanoid algorithm, see PMID 25429972.*

Back

How can I see if a protein is conserved?

You can see on your search results species that contain orthologs across the 10 species currently supported by OpenProt (**1**). Species are abbreviated using a two letter code, the first letters of the species and sub-species names (for example, *Rattus norvegicus* is abbreviated RN). The darker the

blue colour, the more similar is the protein sequence from the ortholog in that species. All details for identified orthologs and paralogs can be found under the **details** tab.

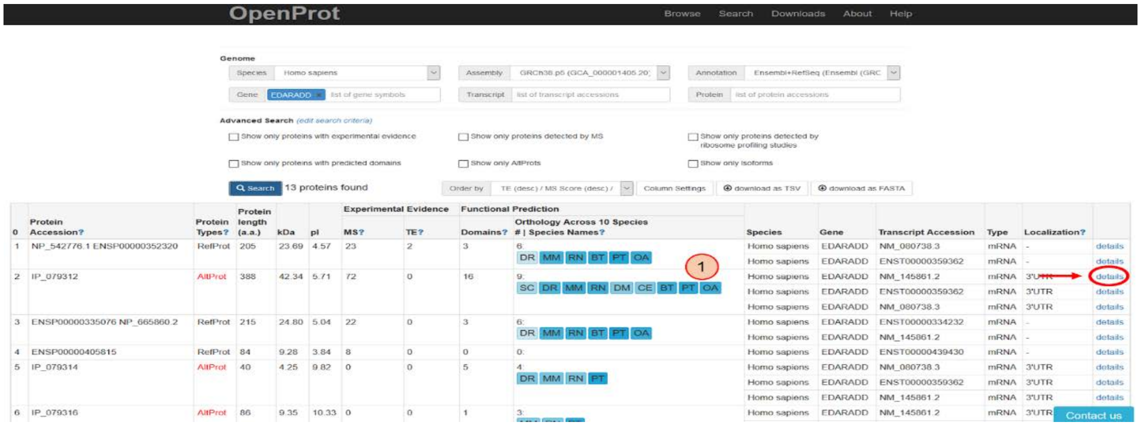

Then, you can click on the **conservation** tab, that will display orthologs and paralogs. The number on the Conservation tab corresponds to the number of species with at least one identified ortholog, out of the 10 species currently supported by OpenProt.

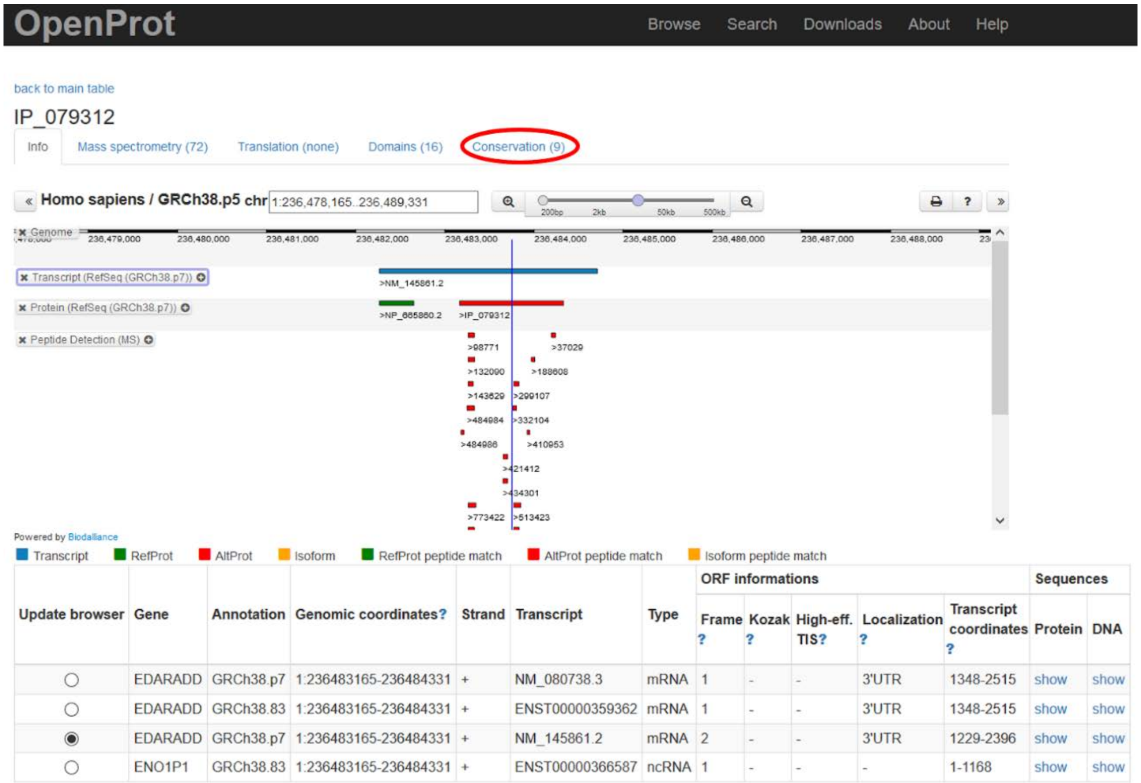

The tree of orthologs and paralogs is then displayed. Orthologs and paralogs are separated in two trees (yellow node, **1**). Then, orthologs for each species can be displayed or hidden by clicking on the species node (**2**). The size of the nodes relates to the number of orthologs, when the colour relates to the homology (identity percentage, **3**).

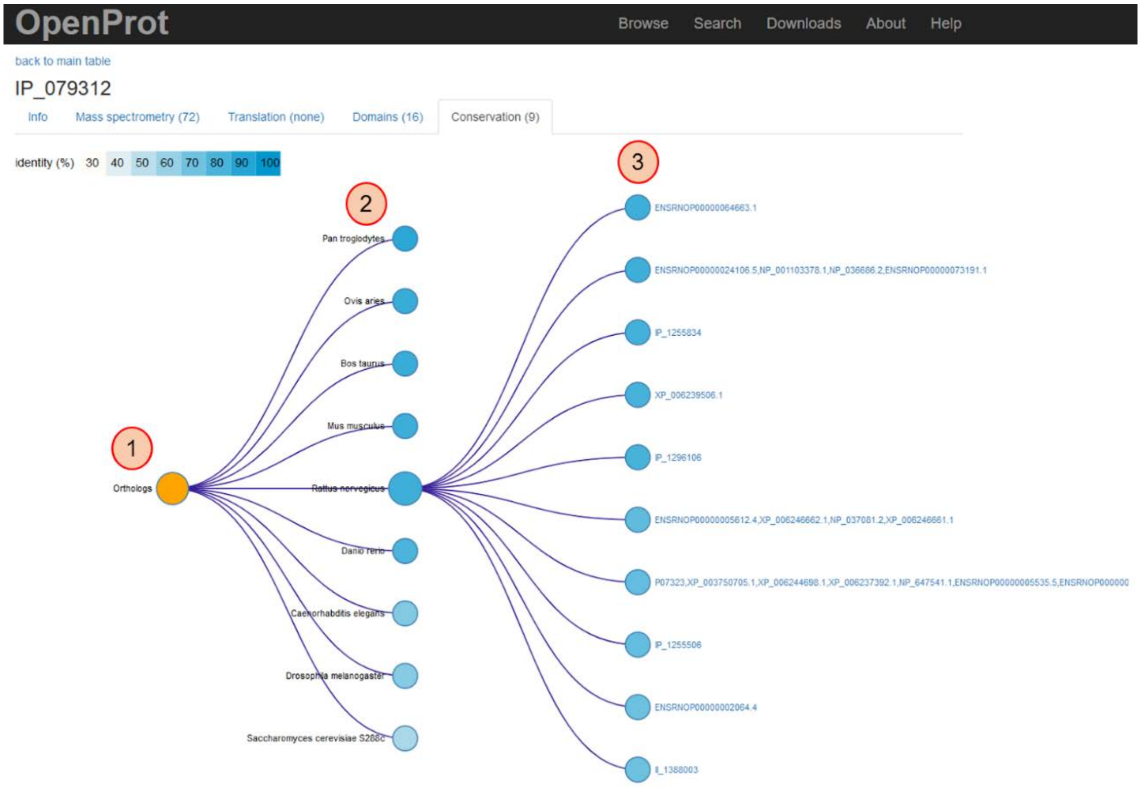

Details for each identified ortholog pair and paralog pair can be displayed by clicking on the **accession** key.

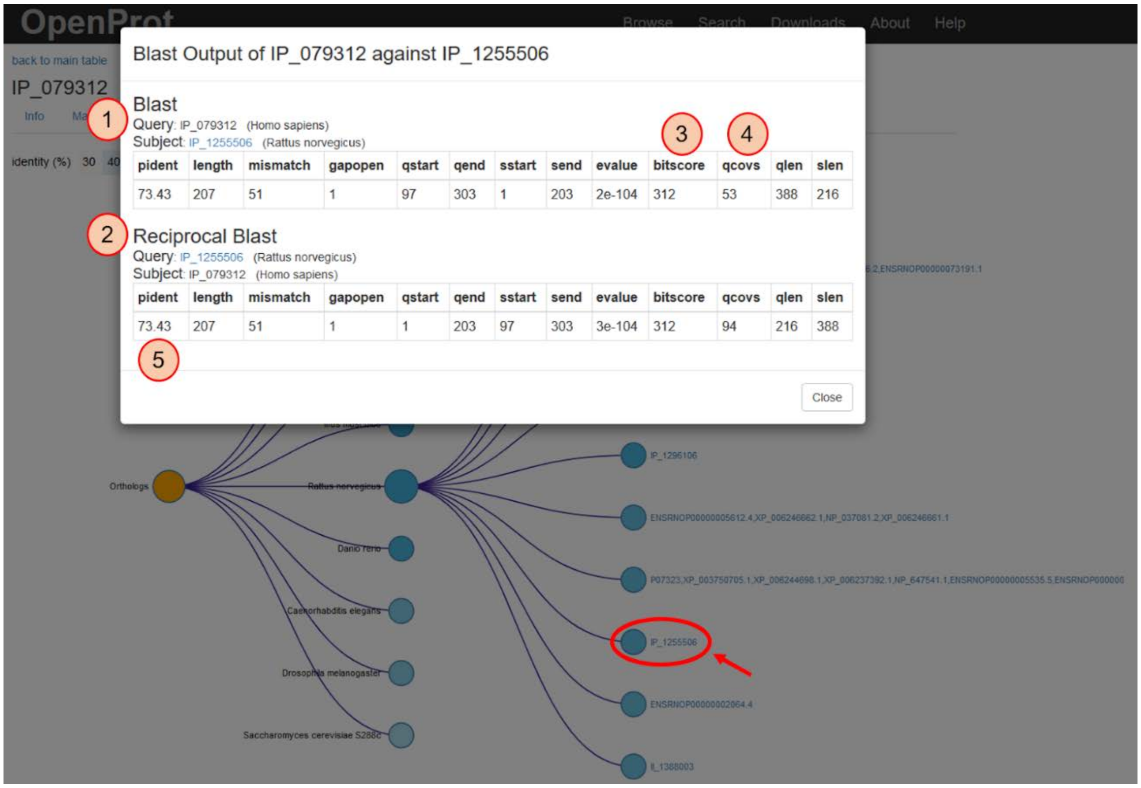

Both the BLAST (**1**) and reciprocal BLAST (inverted query species, **2**) results are displayed. Notably of interest, the bitscore (**3**) and the query sequence coverage (qcovs, **4**) are displayed. The identity percentage is indicated as well (pident, **5**). Finally, by clicking on the blue marked accession of the identified ortholog from the pop-up window, one can directly access the details tab to that specific protein (in the above example IP_1255506 in *Rattus norvegicus*).

Back

I found a novel protein but it is weakly conserved, is it a random ORF?

A **lack of conservation does not necessarily mean the ORF is random**. Several studies showed that *de novo* genes rise up from short ORFs (PMID 29556078) not conserved across species. Furthermore, transcriptome annotation is more thorough in human than in other species. This can lead to an apparently weakly conserved sequence, which is in fact due to poorer transcriptome annotations in other species.

Back

Conservation analysis truths: recommendations

AltProts have a **median length of 45 amino acids**, much shorter than the 460 amino acids of RefProts. This is to keep in mind when looking at orthology, and this directed our choice of threshold (see above). It is possible one ortholog passes the filter when the homology rely only on a conserved functional domain. However, such cases would always have a bitscore close to the threshold of 40. That is why we always encourage users to **look at the protein sequences, their alignment and scores**. These can be found by clicking on the accession key of identified orthologs and/or paralogs (see above).
OpenProt currently does not account for **outparalogs**. For example, should a gene undergo a duplication event in a distant species, all protein sequences derived from the original and the "copy" genes will be identified as orthologs from another species when they actually make up separate ortholog groups. That is why we encourage for **careful exploration of OpenProt** conservation data for each candidate of interest.

Back
